# Whole-exome sequencing of muscle-invasive bladder cancer identifies recurrent copy number variation in IPO11 and prognostic significance of importin-11 overexpression on poor survival

**DOI:** 10.18632/oncotarget.12315

**Published:** 2016-09-28

**Authors:** Junjie Zhao, Weidong Xu, Minghui He, Zhensheng Zhang, Shuxiong Zeng, Chong Ma, Yinghao Sun, Chuanliang Xu

**Affiliations:** ^1^ Department of Urology, Yantai Yuhuangding Hospital, Yantai 264000, China; ^2^ Department of Urology, Changhai Hospital, The Second Military Medical University, Shanghai 200433, China; ^3^ Cancer Research Department, BGI-Shenzhen, Yantian District, Shenzhen, Guangdong 518083, China

**Keywords:** bladder cancer, tumor progression, IPO11, prognosis, whole-exome sequencing

## Abstract

Non-muscle-invasive bladder cancer (NMIBC) often has a worse prognosis following its progression to muscle-invasive bladder cancer (MIBC), despite radical cystectomy with pelvic lymph node dissection combined with chemotherapy. Therefore, the discovery of novel biomarkers for predicting the progression of this disease and of therapeutic targets for preventing it is crucial. We performed whole-exome sequencing to analyze superficial tumor tissues (T_sup_) and basal tumor tissues (T_bas_) from 3 MIBC patients and identified previously unreported copy number variations in IPO11 that warrants further investigation as a molecular target. In addition, we identified a significant association between the absolute copy number and mRNA expression of IPO11 and found that high importin-11 expression was correlated with poor 3-year overall survival (OS), cancer-specific survival (CSS) and cancer-free survival (CFS) compared with low expression in the BCa patients. Importin-11 overexpression was also an independent risk factor for CSS and CFS in the BCa patients. Our study has revealed that IPO11 copy number amplification contributes to its overexpression and that these changes are unfavorable prognostic factors in NMIBC. Thus, IPO11 copy number amplification and importin-11 overexpression are promising biomarkers for predicting the progression and poor prognosis of patients with NMIBC.

## INTRODUCTION

Bladder cancer (BCa) is the most prevalent urinary malignancy in China, with incidence and mortality rates of 80.5 and 32.9 per 100,000, respectively, in 2015 [1]. Among newly diagnosed patients, approximately 70%-80% present with non-muscle-invasive BCa (NMIBC). NMIBC is generally managed by transurethral tumor resection, a minimally invasive surgical treatment. NMIBC patients have a good prognosis if the disease has not progressed to muscle-invasive BCa (MIBC), and NMIBC eventually progresses to MIBC in approximately 30% of patients. Radical cystectomy with pelvic lymph node dissection is the standard treatment option for local MIBC, but approximately 50% of patients still develop local recurrence within two years. The 3-year survival rate is less than 50% [2]. However, the molecular mechanisms underlying progression from NMIBC to MIBC are still poorly understood, and knowledge of the molecular biomarkers that predict the risk of clinical progression of NMIBC, as well as the targeted drugs that block this progression, is still lacking. Therefore, studies of the molecular mechanisms of BCa invasion and progression are necessary.

However, cancer progression in humans is a multistep process, and it is difficult to acquire routine tumor samples from patients at multiple disease stages for basic research purposes. Recent advances in intratumoral heterogeneity (ITH) research achieved using next-generation sequencing (NGS) approaches to assess various types of human cancers have provided the unique opportunity to study the molecular mechanisms of human tumor progression by comparing multiple tumor cell subpopulations from different anatomic locations [3, 4]. Tumor-specific somatic DNA alterations in BCa have been identified through whole-exome sequencing (WES) by comparing gene expression in tumor tissues with that in the corresponding normal germline in matched blood or adjacent normal bladder tissues [5]. However, spatial ITH in BCa has not been well studied.

In this study, we focused on characterization of somatic DNA alterations between basal invasive MIBC tumors and the corresponding superficial tumor parts invading the bladder lumen from 3 patients without a history of neoadjuvant chemotherapy to determine the molecular mechanisms of tumor progression and the potential molecular signatures associated with clinical outcomes in BCa.

## RESULTS

The average sequencing depth was 130×, and 92.7% of the target regions were covered by a depth of at least 10×. We compared superficial tumor samples with normal samples, basal tumor samples with normal samples, and basal tumor samples with superficial tumor samples, respectively. A total of 905, 728, and 29 candidate somatic mutations were detected in the three comparisons, respectively, including 429 synonymous, 1066 missense, 112 nonsense, 30 splicing, 18 frameshift and 7 in-frame mutations, resulting in averages of 302, 243, and 10 somatic mutations, respectively, in exonic regions. The predominant substitution in these mutations was a C:G > T:A transversion.

The comparison of the superficial tumor tissues with the normal tissues resulted in the identification of four recurrently mutated genes with functional mutations (missense, nonsense, splicing, small insertion or deletion): MLL2, SUGP2, TP53 and TTN. Four genes (AKAP9, ATM, CREBBP, and NF1) were mutated in only one subject but were reported on the Cancer Gene Census list, and the mutations were predicted to be detrimental by SIFT. Kyoto encyclopedia of genes and genomes (KEGG) enrichment analysis of the genes with functional mutations revealed that 15 pathways were significantly enriched and that the largest number of mutated genes was associated with the Notch signaling pathway (Table 1).

**Table 1:** KEGG enrichment analysis of the genes with functional mutations

	KEGG pathway	Number of genes in the KEGG pathway	Number of differentially expressed genes	Expected number in the pathway	Ratio of enrichment	Raw *P* value	Adjusted *P* value
T_sup_ vs. Normal	Notch signaling pathway	47	8	0.86	9.33	1.99E-06	0.0003
	Focal adhesion	200	14	3.65	3.84	2.11E-05	0.0009
	Cell cycle	124	11	2.26	4.86	1.82E-05	0.0009
	Long-term potentiation	70	8	1.28	6.26	4.13E-05	0.0014
	Calcium signaling pathway	177	12	3.23	3.72	0.0001	0.0022
	Hypertrophic cardiomyopathy (HCM)	83	8	1.51	5.28	0.0001	0.0022
	Prostate cancer	89	8	1.62	4.93	0.0002	0.0024
	Glioma	65	7	1.19	5.9	0.0002	0.0024
	Amino sugar and nucleotide sugar metabolism	48	6	0.88	6.85	0.0002	0.0024
	p53 signaling pathway	68	7	1.24	5.64	0.0002	0.0024
	Tight junction	132	10	2.41	4.15	0.0002	0.0024
	Metabolic pathways	1130	38	20.62	1.84	0.0003	0.0033
	Starch and sucrose metabolism	54	6	0.99	6.09	0.0004	0.0037
	Pathways in cancer	326	16	5.95	2.69	0.0004	0.0037
	Ribosome biogenesis in eukaryotes	80	7	1.46	4.79	0.0007	0.0061
T_bas_ vs. T_sup_	Adherens junction	73	2	0.04	56.26	0.0006	0.0012
	Tight junction	132	2	0.06	31.12	0.0019	0.0019

No recurrent mutations were detected in the comparison of the basal tumor tissues with the superficial tumor tissues. KEGG enrichment analysis of the genes with functional mutations indicated that 2 pathways were significantly enriched and that the largest number of mutated genes was associated with the adherens junction signaling pathway (Table 1).

The exome sequencing data were examined to characterize the BCa somatic copy number alterations, revealing that 220, 274, and 744 genes were deleted in the superficial tumor tissues relative to the normal tissues, in the basal tumor tissues relative to the normal tissues, and in the basal tumor tissues relative to the superficial tumor tissues, respectively, and that 2669, 1667, and 1235 genes were amplified. Finally, the comparison of the basal tumor tissues with the superficial tumor tissues revealed that 24 genes were amplified and that 44 were deleted in two cases; in addition, 1211 and 700 genes were altered in only one subject.

**Figure 1:**
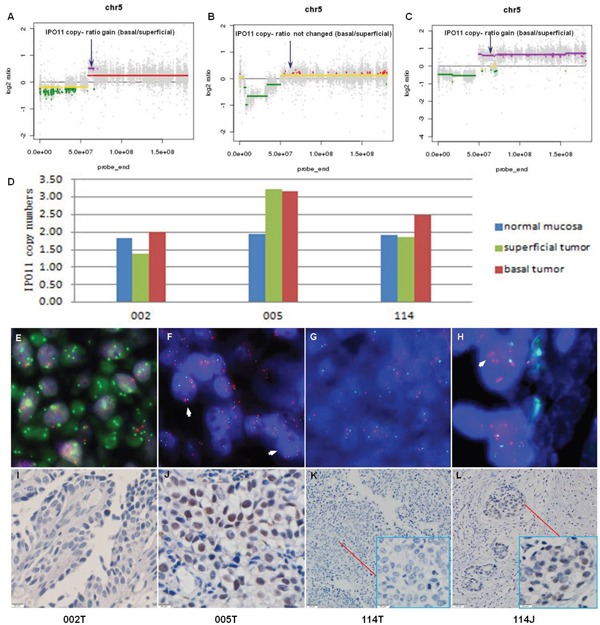
Recurrent copy number variation in IPO11 identified by whole-exome sequencing and confirmed by qPCR and FISH Copy number ratio (T_bas_ vs. T_sup_) determined by whole-exome sequencing of samples from 3 MIBC patients (sample IDs: 002, 005, and 114; T: superficial tumor, and J: basal tumor); the arrows indicate the locations of IPO11. **A.** The IPO11 copy number ratio was increased in sample 002; **B.** this ratio was unchanged in sample 005; **C.** and this ratio was increased in sample 114. IPO11 copy number variations were confirmed in samples 002, 005 and 114 by quantitative polymerase chain reaction **D.** and fluorescence *in situ* hybridization **E-H.** The orange fluorescent spot indicates the IPO11 gene (5q12.1), the green fluorescent spot represents the AHRR gene (5p15.3), and the arrowhead indicates the nucleus with an increased IPO11 copy number. The microscopic data from immunohistochemical analysis of IPO11 are presented at the bottom of the figure **I-L.**

**Figure 2:**
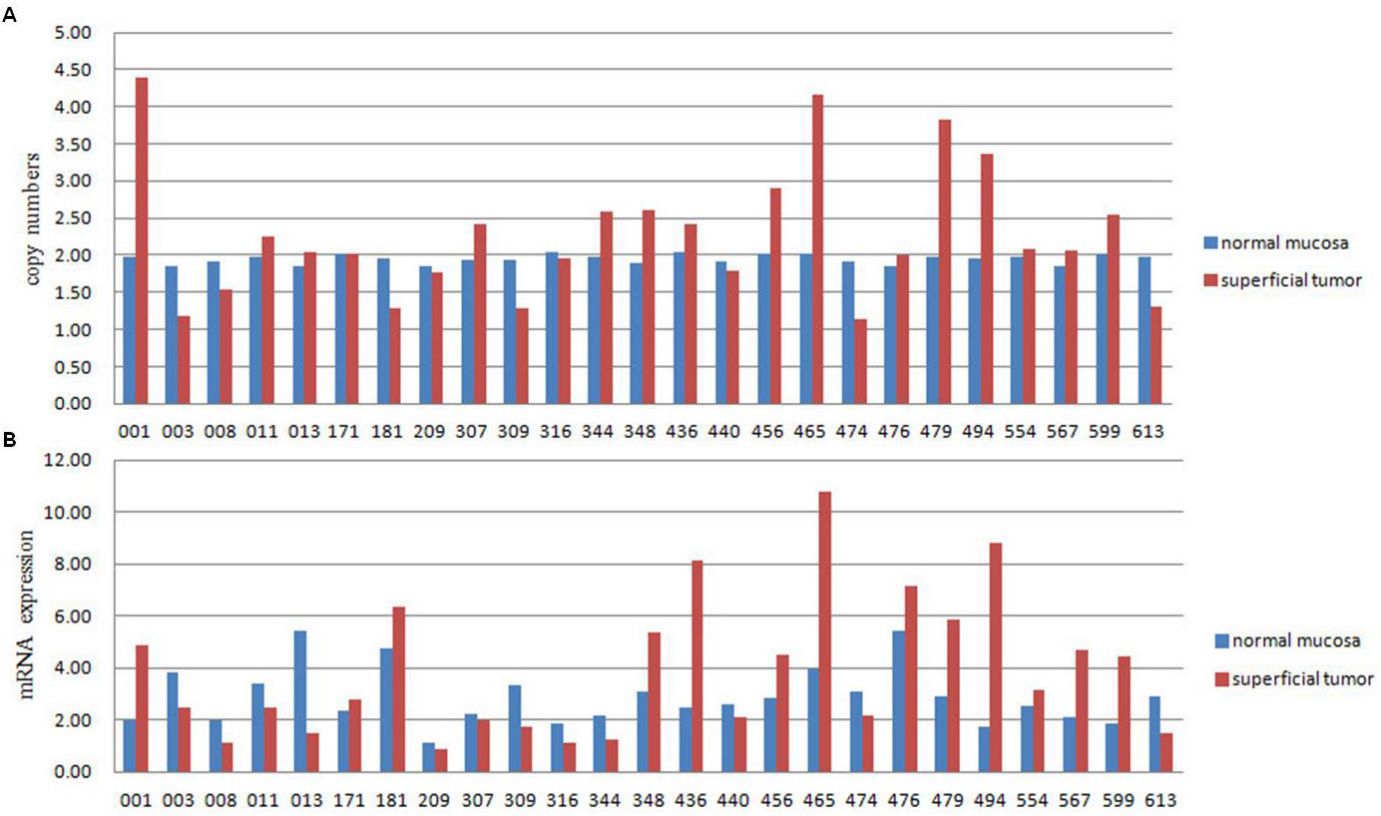
Absolute copy number is significantly correlated with IPO11 mRNA expression Absolute copy number **A.** and mRNA expression **B.** of IPO11 were detected in 25 BCa cases by quantitative polymerase chain reaction. The copy number was considered increased or decreased when the absolute copy number was greater than 2.4 or less than 1.6, respectively. High or low mRNA expression was assumed when expression in the tumor was more or less than 50% of that in the normal mucosa, respectively. The kappa consistency test was performed to assess the relationship between the absolute copy number and mRNA expression, and it revealed a significant association (*P* < 0.0001, kappa value = 0.578).

Among the 24 genes that were amplified in two cases, IPO11 has not been previously reported to be associated with cancer. Notably, further analyses of samples from the 3 sequenced cases, including molecular confirmation of copy number variations (CNVs) and CNV detection by fluorescence *in situ* hybridization (FISH), confirmed the sequencing results in all cases and revealed that the absolute IPO11 copy numbers were actually altered in all 3 cases in the superficial tumors or basal tumors or both. The immunohistochemistry (IHC) findings showed that overexpression of importin-11, encoded by IPO11, was associated with increased IPO11 copy numbers in the sequenced tissues (Figure 1). Hence, we further analyzed the absolute copy numbers and mRNA expression of IPO11 in additional tumor tissues from 25 BCa patients (a superficial tumor was used if the tumor was invasive) and matched normal mucosa by quantitative polymerase chain reaction (qPCR). Increased and decreased IPO11 copy numbers were detected in 10 and 6 tumors, respectively. The absolute copy numbers were determined by qPCR, which revealed that the IPO11 CNV rate was 64.0% (16/25) and that IPO11 copy number alterations were significantly associated with aberrant expression of IPO11 mRNA in the bladder tumors (Figure 2).

**Figure 3:**
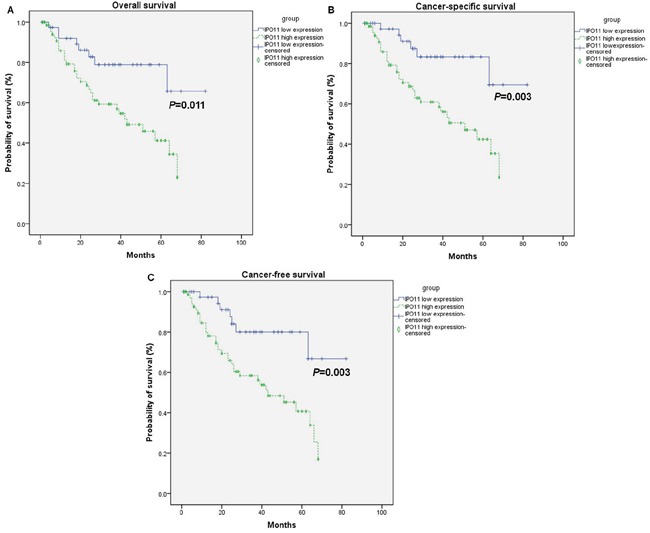
Overexpression of importin-11 predicts poor survival of BCa patients The overall survival **A.**, cancer-specific survival **B.**, and cancer-free survival **C.** rates of 114 BCa patients undergoing radical cystectomy with pelvic lymph node dissection, grouped according to the importin-11 expression levels (high expression vs. low expression).

In an expanded sample of 114 BCa patients with follow-up data, those with importin-11 overexpression had worse overall survival (OS), cancer-specific survival (CSS) and cancer-free survival (CFS), as determined using a Kaplan-Meier analysis curve (Figure 3). Multivariate Cox proportional hazards regression analysis was also performed with adjustments for the pT and pN stages to account for the known prognostic factors associated with the pT stage and nodal status. The results demonstrated that importin-11 overexpression remained significantly associated with reductions in CSS (*P* = 0.018, hazard ratio of 3.191, 95% CI: 1.222 to 8.335) and CFS (*P* = 0.015, hazard ratio of 2.972, 95% CI: 1.235 to 7.150) (Table 2).

**Table 2:** Cox regression analysis of parameters potentially influencing cancer-specific survival and cancer-free survival in 114 patients with urothelial carcinoma of the bladder

Parameters	Cancer-specific survival			Cancer-free survival		
	HR	95% CI	*P*	HR	95% CI	*P*
Univariate Cox regression analysis						
Age (≥ 65 vs. < 65)	1.027	0.992-1.064	0.131	1.031	0.997-1.066	**0.077**
Sex (male vs. female)	0.641	0.265-1.551	0.324	0.707	0.294-1.698	0.438
Tumor stage (T_2_-T_4_ vs. Tis-T_1_)	1.616	0.735-3.552	0.232	1.375	0.671-2.820	0.384
Tumor grade (high vs. low)	2.458	0.335-18.053	0.377	1.331	0.318-5.565	0.695
Lymphatic invasion (yes vs. no)	3.493	1.678-7.271	**0.001**	3.137	1.523-6.460	**0.002**
Lymph node metastasis (yes vs. no)	3.119	1.533-6.347	**0.002**	2.768	1.376-5.565	**0.004**
IPO11 expression (high vs. low)	3.891	1.515-9.992	**0.005**	3.537	1.487-8.415	**0.004**
Multivariate Cox regression analysis*						
Age (≥ 65 vs. < 65)	-	-	-	1.035	0.999-1.072	0.056
Lymphatic invasion (yes vs. no)	2.039	0.803-5.179	0.134	1.942	0.820-4.598	0.131
Lymph node metastasis (yes vs. no)	1.621	0.654-4.017	0.297	1.682	0.724-3.909	0.227
IPO11 expression (high vs. low)	3.191	1.222-8.335	**0.018**	2.972	1.235-7.150	**0.015**

Abbreviations: HR, hazard ratio; CI, confidence interval.

* multivariate analysis of cancer-specific and cancer-free survival included only those parameters with a *P* value of ≤0.10 in univariate analysis.

## DISCUSSION

The progression of NMIBC is strongly associated with worse survival outcomes; however, the molecular mechanisms underlying its progression remain unclear. ITH has been frequently detected in various cancer types and has been reported to influence pathways of cancer progression [6–8]. However, it was not until very recently that a small number of groups elucidated the intratumoral genetic heterogeneity of several cancer types using NGS approaches with DNA extracted from primary tumors [9, 10]. Genomic variation can be identified and cancer progression pathways can be inferred by comparing multiple tumors from different anatomic locations [5, 11]. A recent study has described intratumoral spatial heterogeneity in a single patient that may be reflective of cancer evolutionary dynamics [12]. BCa tumors are highly heterogeneous; however, intratumoral spatial heterogeneity has rarely been reported in MIBC. We postulate that basal tumors rooted in the muscular layer have more aggressive molecular phenotypes than the corresponding superficial tumors growing into the bladder lumen. The genomic differences detected between basal and superficial tumors likely reflect the early hallmarks of BCa progression. Our WES results demonstrated distinct differences between the basal and superficial tumors, either in terms of single-nucleotide variations (SNVs) or structural variations, reflecting the characteristic features of ITH in MIBC. ITH arises in different ways; it may occur at the beginning of the carcinogenesis process, or it may result from development of a different phenotype halfway through this process [5]. Hence, the genomic alterations present in basal tumors could promote cancer invasion and shed light on the molecular mechanisms of tumor progression.

In our exome sequencing analysis, we detected considerable genomic differences among the MIBC tissues from different tumor locations. For example, we identified four genes (MLL2, SUGP2, TP53 and TTN) with recurrent functional mutations in the comparison of superficial tumor tissues with normal tissues. MLL2 is a histone methyltransferase that is part of a large protein complex, ASCOM, that regulates transcription of the β-globin and estrogen receptor genes [13], and recent evidence indicates that MLL2 mutations result in genomic instability, which is a strong driver of tumorigenesis [14]. In our study, we found that 2/3 subjects harbored a frameshift deletion in MLL2, indicating that MLL2 probably also plays a role in tumorigenesis in BCa. SUGP2 is a member of the arginine/serine-rich family of splicing factors and functions in mRNA processing [15]. It has been shown to bind to the intronic region of the iron-sulfur cluster assembly gene (ISCU), which is affected by the hereditary myopathy with lactic acidosis (HML) mutation [16]; however, no previous report has described involvement of SUGP2 in tumorigenesis. In this study, we found two missense mutations in SUGP2 in two subjects, and they were predicted to be damaging by SIFT software. These results indicate that abnormal mRNA processing may contribute to carcinogenesis in BCa. TP53 is a well-known tumor suppressor gene that induces growth arrest or apoptosis, depending on the cell type and physiological conditions. Mutations in this gene play a key role in BCa progression and are also associated with an increased cancer risk [17]. We detected two TP53 missense mutations in two different subjects that were predicted to be deleterious by SIFT software. TP53 likely functions abnormally in BCa tumorigenesis. TTN is one of the longest genes examined in this study; thus, the two mutations detected in this gene probably occurred randomly. On the other hand, no recurrent mutations were identified in the comparison of basal tumor tissues with superficial tumor tissues.

KEGG pathway analysis of the functional mutations revealed the different signaling pathways affected in the different comparisons. The Notch signaling pathway was the most significantly enriched pathway in the comparison of superficial tumor tissues with normal tissues. These results are in agreement with those of Rampias' study [18]. In addition, the adherens junction pathway was significantly enriched in the comparison of basal and superficial tumor tissues, reflecting differences in the molecular mechanisms involved in the development versus progression of BCa; thus, mutations of genes in adherens junction pathway may play important roles in tumor progression.

In addition to functional mutations, CNVs also affect gene functions. In our study, the incidence of CNVs was significantly higher than that of mutations in the comparisons between the different tumor sites; however, the functional roles of the CNVs were unclear. Our analysis focused on CNVs in IPO11, due to not only the high frequency but also the potential associations with tumorigenesis and progression.

Importin-11, a 116-kD protein encoded by IPO11, is a member of the karyopherin family. These proteins mediate the nucleocytoplasmic transport of proteins and nucleic acids through nuclear pore complexes in a Ran-dependent manner [19]. The karyopherin family includes more than 20 members that can be classified as importins and exportins, which facilitate nuclear import and export, respectively. The classical nuclear import pathway is mediated by a heterotrimer consisting of importin-β, importin-α and cargo. Importinα acts as an adaptor molecule, interacting with the nuclear localization signal (NLS) of cargo and importin-β at the respective specific binding domain [20]. It has been gradually realized that various tumor suppressors and oncoproteins are regulated through the nuclear transport pathway and that deregulation of the nuclear transport machinery plays central roles in cell proliferation and transformation and tumor progression [21]. Overexpression of importin-α1 (also known as karyopherin-α2) has been frequently reported to play a role in carcinogenesis and to contribute to poor clinical outcomes in multiple cancer types. Some studies have considered that importin-α1 overexpression is an independent risk factor for survival and a potential therapeutic target for cancer [22, 23]. However, mammalian cells contain only a small number of karyopherin-α proteins and a larger number of different karyopherin-β proteins, and many karyopherin-β molecules can transport cargo independent of karyopherin-α by directly binding to the NLS of the cargo. Moreover, the direct transport of importin-β•cargo is faster and more efficient than the transport of cargo mediated by an importin-α•β heterodimer. Each karyopherin-β molecule has its own specific cargo, including tumor progression-related factors [24]. Thus, karyopherin-β shows promise target in inhibiting tumor development and progression. However, only a limited number of studies have reported karyopherin-β1 overexpression in cancer [25], and the main features of its inherent mechanism of regulation and the functional roles of other members of the karyopherin-β family remain unknown.

In this study, we detected a high frequency of IPO11 CNVs by WES, and this result was confirmed by qPCR and FISH analyses. Importantly, high importin-11 expression was significantly associated with reduced 3-year OS, CSS, and CFS rates, and it was also an independent risk factor for CSS and CFS in the BCa patients, indicating that high expression of this gene stimulates BCa progression and that the CNV in IPO11 is a crucial component of the molecular mechanism of its overexpression.

The identity of the cargo transported by importin-11 is currently poorly understood. Importin-11 directly binds to UbcM2 to promote UbcM2 transport from the cytoplasm into the nucleus [19]. UbcM2 is an important participant in the ubiquitin cascade, and a variety of tumor suppressors are degraded mainly through the ubiquitin degradation pathway. Tumor progression may be caused by importin-11 overexpression through acceleration of the degradation of some tumor suppressors via the promotion of UbcM2 nuclear translocation. Moreover, recent studies have shown that UbcM2 also abolishes the stability and activity of Nrf2, which has been shown to be associated with tumor progression [26]. It is not very clear whether importin-11 transports other oncogenes and tumor suppressor genes; hence, its roles in tumorigenesis and progression require further study.

In conclusion, we have identified novel and frequent somatic CNVs in IPO11 in MIBC. We have also revealed significant associations of an increased IPO11 copy number and importin-11 overexpression with worse clinical outcomes of BCa patients. These results, if validated, could be applied in potential prognostic algorithms to aid in clinical decision making in the treatment of patients with this difficult disease.

## MATERIALS AND METHODS

### Sample collection and processing

Three patients (sample IDs: 002, 005 and 114) with chemotherapy-naïve MIBC (WHO 2004 stages IV, III and II, respectively) were treated by radical cystectomy. DNA was extracted from superficial tumor tissues (T_sup_) and basal tumor tissues (T_bas_) separated from the bladder mucosa, as well as from matched normal bladder mucosa located distal to the tumors (a distance of over 3 cm), for sequencing. Information on the patients and tumor tissues is presented in Table 3.

**Table 3:** Clinical and pathologic characteristics of 3 patients assessed by whole-exome sequencing

	Sample ID		
	002	005	114
Patient age (at surgery)	78	59	50
Gender	Male	Male	Male
Race	Chinese	Chinese	Chinese
Smoking history	NO	NO	30 years, 20 cigarettes/d, current smoker
Occupation	Peasant	Civil servant	Peasant
Pathologic T	T_4a_	T_3b_	T_2b_
Pathologic N	N_1_	N_0_	N_0_
Distant metastasis	NO	NO	NO
Histologic type	Urothelial carcinoma	Urothelial carcinoma	Urothelial carcinoma
Histologic grade	High grade	High grade	High grade
Multiple tumors	YES	YES	YES
Size of largest tumor (cm)	4×3×1.5	3×2×2	4.5×4×1
Tumor phenotype	Solid	Cauliflower-like	Solid
Concurrent carcinoma *in situ*	NO	NO	NO
Recurrent tumor	YES	NO	NO
Adjuvant chemotherapy	NO	NO	NO
Surgical procedure	ORC	ORC	LRC
Type of urinary diversion	Ileal conduit	Ileal conduit	Ileal conduit
Date of surgery	2011.12.19	2011.12.30	2012.4.11
Follow-up time (months)	29	35	31
Results of follow-up	Osseous metastasis 6 months after operation	No recurrence or distant metastasis was detected	Osseous metastasis 12 months after operation
Survival of patient (2015.6.30)	Died (2014.5.19)	Survived	Survived

Abbreviations: ORC, open radical cystectomy; LRC, laparoscopic radical cystectomy.

A total of 114 BCa samples were obtained from patients undergoing cystectomy at Changhai Hospital, the Second Military Medical University (Shanghai, China), between 2009 and 2013 for further verification. Follow-up data were available for each patient. Tissue samples from primary tumors were collected and prepared for IHC staining. Fresh-frozen tissues from 25 BCa patients and matched normal bladder tissues were also collected and immediately snap-frozen in liquid nitrogen within 10 minutes after collection for RNA and DNA analyses. All tissue samples were reviewed by an attending genitourinary oncology pathologist for verification of the qualifications of the tumor and normal tissues, particularly to ensure for the presence of a sufficient percentage of tumor nuclei in the tumor samples and for the absence of tumor cells in the normal control samples. All sample collection and experimental procedures were conducted with approval of the Institutional Review Board of Changhai Hospital.

### WES

Genomic DNA (gDNA) was isolated from superficial and basal bladder tumors and matched normal bladder tissues from 3 patients for WES. Exome capture was conducted using an Agilent SureSelect All Exon 50 Mb capture library (Agilent Technologies, Santa Clara, CA, USA). Briefly, each qualified gDNA sample was randomly broken into fragments with a maximum length of 150 to 200 bp, and then adapters were ligated to both ends of the resulting fragments. The adapter-ligated templates were purified using Agencourt AMPure SPRI beads (Beckman Coulter Inc., Brea, CA, USA), and fragments with an insert size of approximately 200 bp were excised. Extracted DNA was amplified by ligation-mediated polymerase chain reaction (LM-PCR), purified, and hybridized to a SureSelect Biotinylated RNA Library (BAITS) for enrichment. Hybridized fragments were bound to streptavidin beads, whereas non-hybridized fragments were washed out after 24 h. Captured LM-PCR products were assessed with an Agilent 2100 Bioanalyzer to estimate the magnitude of enrichment. Each captured library was then loaded onto a HiSeq 2000 platform (Illumina, San Diego, CA, USA), and high-throughput sequencing was performed independently for each library to ensure that the desired average fold coverage was achieved for each sample. Raw image files were processed using Illumina base calling software 1.7 for base calling with the default parameters, and 90-bp paired-end reads were obtained for each sample.

### Exome capture, read mapping and variation detection

The adapter sequences were removed from the raw reads, and low-quality reads with more than 5 Ns and low-quality bases were discarded. Then, high-quality reads were gap aligned to the NCBI human reference genome (hg19) using Burrows-Wheeler Aligner (BWA v0.5.9) [27] with the default parameters. Local realignment of the original BWA alignment was performed using Genome Analysis Toolkit (GATK v1.0.6076) [28], and then Picard was used to mark duplicate reads.

Somatic substitutions were detected with VarScan2.2.5 [29] based on the BWA alignment, and high-confidence somatic SNVs were called if the following criteria were met: (I) sufficient coverage (≥ 10×) at the genomic position for both the tumor and normal samples; (II) presence of the variant in at least 10% of the total reads from the tumor tissues and less than 2% of those from the normal tissues; (III) presence of the variant in at least three reads from the tumor tissues; and (IV) a distance between adjacent somatic SNVs of over 10 bp.

High-confidence somatic insertions and deletions (indels) were called using the following steps: (I) candidate somatic indels were predicted with GATK Somatic Indel Detector with the default parameters; (II) for each predicted somatic indel, local realignment was performed with combined normal and tumor BWA alignment files; and (III) high-confidence somatic indels were defined after the filtering of germline events.

All high-confidence somatic mutations were filtered out using dbSNP (version 135), which contains common polymorphisms with no known medical impacts. The remaining mutations were annotated with ANNOVAR [30] and subjected to subsequent analyses.

### Copy number analysis

We obtained the exonic regions enriched by exome sequencing from Agilent. These regions were extended by 90 bp (the length of each read) to the left and right, and overlapping regions were merged. Regions of chromosome Y, as well as telomeric and centromeric regions (obtained from UCSC), were removed. As a result, 187,308 exonic regions remained, which were merged with overlapping regions. Then, CNVs were identified with Exome CNV [31] by comparing the coverage depths of the tumor and matched normal samples after normalizing by the mean coverage depth of each exome.

### CNV molecular confirmation

gDNA was isolated by phenol-chloroform extraction after freezing of the tissues in nitrogen. Copy number verification of IPO11 in 5q12.1 was performed using real-time qPCR for the tissue samples from the 3 sequenced patients and an additional 25 BCa patients who had undergone radical cystectomy. The following three primer pairs were designed to amplify regions covering the full length of IPO11, from the C-terminus to the N-terminus, referred to as P1 to P3, using NCBI's primer designing tool Primer-Blast (http://www.ncbi.nlm.nih.gov/tools/primer-blast/): P1 (forward, TCCCAGACAGTGGCCTGAACT and reverse, CAGCAAGTCGTTTAGATGCCAGT), P2 (forward, CGCAGTAGGTCTATGCCAGTCC and reverse, TTCTTGCGAACCATCCAAATGA), and P3 (forward, TAACCCAGCCTGCCGTTTGTA and reverse, GAAGGGCCCCCTCATAAATATAAAA). Three housekeeping genes, POLR2A, POP1 and RPP14, were used as reference genes for normalization of possible variations in DNA concentrations and differences in DNA quality among the samples. qPCR was performed with SYBR^®^ Premix Ex Taq™ (Takara Bio Inc., Shiga, Japan) using an ABI 7900 System (Applied Biosystems, USA). The 2^−ΔΔ^CT method was used to calculate the relative expression of IPO11 compared with that of the reference genes. The absolute copy number of IPO11 was determined using the equation 2×2^−ΔΔ^CT.

### FISH

The samples from the 3 sequenced patients and 25 aforementioned patients were analyzed by FISH using an IPO11/AHRR FISH Probe Kit (Cytotest Inc., Rockville, MD, USA), which included the following 2 hybridization probes: a locus-specific orange probe for the IPO11 gene, located on the long arm of chromosome 5 (5q12.1), and a locus-specific green probe for the AHRR gene, located on the short arm of chromosome 5 (5p15.3). Formalin-fixed and paraffin-embedded tissues were cut serially into 4 μm sections. The sections were then deparaffinized with xylene, dehydrated with ethanol, and incubated in a pretreatment solution containing sodium thiocyanate at 80°C for 10 minutes, followed by incubation in a protease solution at 37°C for 12 minutes. Next, the sections were washed several times with distilled water and dehydrated with ethanol, and they were subsequently incubated with the probes with simultaneous denaturation of tissue DNA at 82°C for 8 minutes, followed by hybridization at 42°C overnight using StatSpin ThermoBrite (Abbott Molecular, Des Plaines, IL, USA). After several posthybridization washes, nuclei were counterstained with DAPI II (4,6-diamidino-2-phenylindole in 1,4-phenylenediamine) and visualized with an Olympus BX51 microscope. Chromosome 5 and the IPO11/AHRR gene copy numbers were assessed at 1000× magnification.

### RNA extraction and real-time qPCR

Total RNA was isolated using a TRIzol RNA Kit (Invitrogen, Carlsbad, CA, USA) and quantified using an Eppendorf Biophotometer (Eppendorf, Hamburg, Germany). Reverse transcription was conducted using a PrimeScript™ RT Reagent Kit with gDNA Eraser (Takara Bio Inc., Shiga, Japan). Real-time qPCR was performed with a SYBR® Premix Ex Taq™ Kit (Takara Bio Inc., Shiga, Japan) using a StepOnePlus™ Real-Time PCR System (Applied Biosystems, Foster City, CA, USA). The following primers were used for quantification of IPO11 mRNA expression: forward, 5′-GGTCCATCCTGGCTCAACAGA-3′ and reverse, 5′-GCAATCCAGTCATAGCCCAGGTA-3′. β-actin was used as a control, with the following primers: forward, 5′-GCACCGTCAAGGCTGAGAAC-3′ and reverse, 5′-TGGTGAAGACGCCAGTGGA-3′.

### IHC

The samples from the 3 sequenced patients and 114 BCa patients with follow-up data were subjected to IHC staining. Two consecutive 4 mm-thick sections were obtained for IHC assays. Importin-11 expression was assessed using a polyclonal rabbit antibody (14403-1-AP; Proteintech, Chicago, IL, USA). Optimal staining was achieved at a 1:50 antibody dilution. Bound primary antibodies were visualized using an Envision Plus system (Dako, Glostrup, Denmark). Importin-11 expression was observed in the nuclei or cytoplasm or both. Evaluation of importin-11 staining included scoring of the staining intensity (0, 1+, 2+ and 3+) and determination of the percentage of positive tumor cells for each slide. A final IHC score was calculated according to these parameters, indicating negative, weak, moderate or strong staining, as described previously [32].

### Statistical analysis

All statistical analyses were performed using SPSS 13.0 software (Chicago, IL, USA). The kappa consistency test was performed to assess the relationship between the absolute copy number and mRNA expression of IPO11. The Kaplan-Meier method was used to determine survival probability, and differences were assessed using the log-rank test. Univariate and multivariate analyses were performed using Cox proportional hazards regression models, and hazard ratios (HRs) for CSS and CFS were calculated. Statistical significance was set at a two-sided *P* of < 0.05.

## References

[1] Chen W, Zheng R, Baade PD, Zhang S, Zeng H, Bray F, Jemal A, Yu XQ, He J (2016). Cancer statistics in China, 2015. CA Cancer J Clin.

[2] Stein JP, Skinner DG (2006). Radical cystectomy for invasive bladder cancer: long-term results of a standard procedure. World J Urol.

[3] Ke HL, Chen M, Ye Y, Hildebrandt MA, Wu WJ, Wei H, Huang M, Chang DW, Dinney CP, Wu X (2013). Genetic variations in micro-RNA biogenesis genes and clinical outcomes in non-muscle-invasive bladder cancer. Carcinogenesis.

[4] Duenas M, Martínez-Fernández M, García-Escudero R, Villacampa F, Marqués M, Saiz-Ladera C, Duarte J, Martínez V, Gómez M, Martín M, Fernández M (2015). PIK3CA gene alterations in bladder cancer are frequent and associate with reduced recurrence in non-muscle invasive tumors. Mol Carcinog.

[5] Zhang J, Fujimoto J, Zhang J, Wedge DC, Song X, Zhang J, Seth S, Chow CW, Cao Y, Gumbs C, Gold KA (2014). Intratumor heterogeneity in localized lung adenocarcinomas delineated by multiregion sequencing. Science.

[6] Lips EH, van Eijk R, de Graaf EJ, Doornebosch PG, de Miranda NF, Oosting J, Karsten T, Eilers PH, Tollenaar RA, van Wezel T, Morreau H (2008). Progression and tumor heterogeneity analysis in early rectal cancer. Clin Cancer Res.

[7] Navin N, Krasnitz A, Rodgers L, Cook K, Meth J, Kendall J, Riggs M, Eberling Y, Troge J, Grubor V, Levy D (2010). Inferring tumor progression from genomic heterogeneity. Genome Res.

[8] Sottoriva A, Spiteri I, Piccirillo SG, Touloumis A, Collins VP, Marioni JC, Curtis C, Watts C, Tavaré S (2013). Intratumor heterogeneity in human glioblastoma reflects cancer evolutionary dynamics. Proc Natl Acad Sci U S A.

[9] Zhang XC, Xu C, Mitchell RM, Zhang B, Zhao D, Li Y, Huang X, Fan W, Wang H, Lerma LA, Upton MP (2013). Tumor evolution and intratumor heterogeneity of an oropharyngeal squamous cell carcinoma revealed by whole-genome sequencing. Neoplasia.

[10] Mroz EA, Tward AD, Pickering CR, Myers JN, Ferris RL, Rocco JW (2013). High intratumor genetic heterogeneity is related to worse outcome in patients with head and neck squamous cell carcinoma. Cancer.

[11] Gerlinger M, Horswell S, Larkin J, Rowan AJ, Salm MP, Varela I, Fisher R, McGranahan N, Matthews N, Santos CR, Martinez P (2014). Genomic architecture and evolution of clear cell renal cell carcinomas defined by multiregion sequencing. Nat Genet.

[12] Shi JY, Xing Q, Duan M, Wang ZC, Yang LX, Zhao YJ, Wang XY, Liu Y, Deng M, Ding ZB, Ke AW (2016). Inferring the progression of multifocal liver cancer from spatial and temporal genomic heterogeneity. Oncotarget.

[13] Morin RD, Mendez-Lago M, Mungall AJ, Goya R, Mungall KL, Corbett RD, Johnson NA, Severson TM, Chiu R, Field M, Jackman S (2011). Frequent mutation of histone-modifying genes in non-Hodgkin lymphoma. Nature.

[14] Kantidakis T, Saponaro M, Mitter R, Horswell S, Kranz A, Boeing S, Aygün O, Kelly GP, Matthews N, Stewart A, Stewart AF, Svejstrup JQ (2016). Mutation of cancer driver MLL2 results in transcription stress and genome instability. Genes Dev.

[15] Sampson ND, Hewitt JE (2003). SF4 and SFRS14, two related putative splicing factors on human chromosome 19p13.11. Gene.

[16] Nordin A, Larsson E, Holmberg M (2012). The defective splicing caused by the ISCU intron mutation in patients with myopathy with lactic acidosis is repressed by PTBP1 but can be derepressed by IGF2BP1. Hum Mutat.

[17] Kato S, Lippman SM, Flaherty KT, Kurzrock R (2016). The conundrum of genetic “drivers” in benign conditions. J Natl Cancer Inst.

[18] Rampias T, Vgenopoulou P, Avgeris M, Polyzos A, Stravodimos K, Valavanis C, Scorilas A, Klinakis A (2014). A new tumor suppressor role for the notch pathway in bladder cancer. Nat Med.

[19] Plafker SM, Macara IG (2000). Importin-11, a nuclear import receptor for the ubiquitin-conjugating enzyme, UbcM2. EMBO J.

[20] Soniat M, Chook YM (2015). Nuclear localization signals for four distinct karyopherin-beta nuclear import systems. Biochem J.

[21] Muqbil I, Wu J, Aboukameel A, Mohammad RM, Azmi AS (2014). Snail nuclear transport: the gateways regulating epithelial-to-mesenchymal transition?. Semin Cancer Biol.

[22] Alshareeda AT, Negm OH, Green AR, Nolan CC, Tighe P, Albarakati N, Sultana R, Madhusudan S, Ellis IO, Rakha EA (2015). KPNA2 is a nuclear export protein that contributes to aberrant localisation of key proteins and poor prognosis of breast cancer. Br J Cancer.

[23] Zhang Y, Zhang M, Yu F, Lu S, Sun H, Tang H, Peng Z (2015). Karyopherin alpha 2 is a novel prognostic marker and a potential therapeutic target for colon cancer. J Exp Clin Cancer Res.

[24] Chook YM, Süel KE (2011). Nuclear import by karyopherin-βs: recognition and inhibition. Biochim Biophys Acta.

[25] van der Watt PJ, Stowell CL, Leaner VD (2013). The nuclear import receptor Kpnbeta1 and its potential as an anticancer therapeutic target. Crit Rev Eukaryot Gene Expr.

[26] Kanamori M, Higa T, Sonoda Y, Murakami S, Dodo M, Kitamura H, Taguchi K, Shibata T, Watanabe M, Suzuki H, Shibahara I (2015). Activation of the NRF2 pathway and its impact on the prognosis of anaplastic glioma patients. Neuro Oncol.

[27] Li H, Durbin R (2009). Fast and accurate short read alignment with burrows-Wheeler transform. BioInformatics.

[28] McKenna A, Hanna M, Banks E, Sivachenko A, Cibulskis K, Kernytsky A, Garimella K, Altshuler D, Gabriel S, Daly M, DePristo MA (2010). The genome analysis toolkit: a MapReduce framework for analyzing next-generation DNA sequencing data. Genome Res.

[29] Koboldt DC, Zhang Q, Larson DE, Shen D, McLellan MD, Lin L, Miller CA, Mardis ER, Ding L, Wilson RK (2012). VarScan 2: Somatic mutation and copy number alteration discovery in cancer by exome sequencing. Genome Res.

[30] Wang K, Li M, Hakonarson H (2010). ANNOVAR: functional annotation of genetic variants from high-throughput sequencing data. Nucleic Acids Res.

[31] Sathirapongsasuti JF, Lee H, Horst BA, Brunner G, Cochran AJ, Binder S, Quackenbush J, Nelson SF (2011). Exome sequencing-based copy-number variation and loss of heterozygosity detection: ExomeCNV. BioInformatics.

[32] Grupp K, Habermann M, Sirma H, Simon R, Steurer S, Hube-Magg C, Prien K, Burkhardt L, Jedrzejewska K, Salomon G, Heinzer H (2014). High nuclear karyopherin alpha 2 expression is a strong and independent predictor of biochemical recurrence in prostate cancer patients treated by radical prostatectomy. Mod Pathol.

